# Green Campus initiative and its impacts on quality of life of stakeholders in Green and Non-Green Campus universities

**DOI:** 10.1186/s40064-016-1697-4

**Published:** 2016-01-27

**Authors:** Ronnachai Tiyarattanachai, Nicholas M. Hollmann

**Affiliations:** International College, King Mongkut’s Institute of Technology Ladkrabang, Bangkok, Thailand

**Keywords:** Green Campus, Sustainability, Environmental management, Energy and climate change management

## Abstract

In 2010, Universitas Indonesia (UI) developed the UI GreenMetric World University Ranking for universities to share information about their sustainability practices. This ranking system was well aligned with the basis of Sustainability for Higher Education. The scoring system can also be used as a guideline for universities to achieve sustainability in their campuses. Since its first launch, more universities around the world have increasingly participated in the ranking system including many universities in Thailand. This study compared perception of stakeholders in Green Campus and Non-Green Campus universities in Thailand regarding stakeholders’ satisfaction on sustainability practices and perceived quality of life at their campuses. The results showed that stakeholders at the studied Green Campus University were more satisfied and had significantly better perceived quality of life compared to stakeholders from the studied Non-Green Campus university. The results suggested that universities should adopt the criteria set in the UI GreenMetric World University Ranking to achieve better sustainability in their campuses and improve quality of life of their stakeholders.

## Background

The Brundtland Report defined sustainable development as “development that meets the needs of the present without compromising the ability of future generations to meet their own needs” (United Nations 1987). Therefore, it is important to ensure that the world will continue to have sufficient water, materials, and other resources for its living systems. It also means that any development should entail the proper balance of economic, social, and environmental conditions. Since the issue of the Brundtland Report, sustainability has become one of the top concerns of government agencies, companies, and other organizations (USEPA 2015).

For academic institutions, the Stockholm Declaration of 1972 addressed the Sustainability in Higher Education (SHE). The declaration focused on finding ways in which universities, their leaders, lecturers, researchers, and students can engage their resources in responding to the challenges of balancing between the human quest for economic and technological development with environmental preservation. Foo (2013) stated that higher education is a great contributor for society to achieve sustainability. University researchers provided first alarms regarding environmental challenges through their research investigations.

On one point of view, campus expansion has resulted in an increase in the use of motor vehicles and resource consumption (Balsas 2013). Therefore, many universities around the world have attempted to transform their campuses to make them greener. In Malaysia, some efforts were spent to establish a campus greenway network, which provided pleasant condition for walkers, joggers, and cyclists (Foo 2013).

In China, higher education in the country has been expanding at a great pace. By the end of 2011, the total floor area of campus buildings was increased to 780.76 million square meters, which was five times greater than the number in 1998. Subsequently, energy and natural resources were extravagantly consumed to facilitate the expansion. In response to this problem, the first demonstration project of an energy and resource efficient campus was established at Tongji University in 2007. It later successfully drove other Chinese universities to build energy and resource efficient campuses. The implementation of the campus energy management system (CEMS) became an important approach for improving energy and resource consumption efficiency. Still, in 2009, annual energy consumption in universities and colleges was nearly 30 million tons of standard coal equivalent. Water consumption almost reached 4 million tons. These rates of energy consumption and water consumption per student were four times and two times greater than regular Chinese residents (Tan et al. 2014).

Over the past decades, university rankings have become a global phenomenon. They mostly focus on the importance of research and academic reputation, while environmental issues received little or no attention (Alshuwaikhat and Abubakar 2008). Fortunately, Green Campus initiatives have significantly gained momentum since the declaration on SHE (Grindsted and Holm 2012). In 2010, Universitas Indonesia (UI) established the UI GreenMetric World University Ranking as a platform for universities around the world to share their information and practices to achieve sustainability in their campuses. The UI GreenMetric World University Ranking also provided opportunities for each university to examine their strengths and weaknesses in promoting green university and sustainable development (Suwartha and Sari 2013). The ranking method was based on six (6) main categories including setting and infrastructure, energy and climate change, waste management, water usage, transportation, and environmental education. The UI GreenMetric World University Ranking has consistently gained interests from universities around the world since its launch. The number of participating universities increased from 95 universities from 35 countries in 2010 to 360 universities from 62 countries in 2014. In 2014, a total of 15 universities in Thailand participated in the ranking (Universitas Indonesia 2015).

The World Health Organization (WHO) defined the term quality of life (QOL) as an “individual’s perception of their position in life in the context of the culture and value systems in which they live and in relation to their goals, expectations, standards and concerns. It is a broad ranging concept affected in a complex way by the person’s physical health, psychological state, level of independence, social relationships, personal beliefs and their relationship to salient features of their environment” (WHO 1997).

The definition of QOL may vary depending on the context of use. For example, London Mayor Boris Johnson defined sustainable QOL for his Londoners in 2009 as “The quality of life that Londoners experience when living, working, visiting and moving around London is fundamental to how they feel about the city – and to how the capital is perceived from outside. The decisions we make about our city now will shape the quality of life of those who come after us and their view of how successful we have been in our stewardship of the city.” (Higgins et al. 2014).

 Marans (2015) stated that QOL is a multi-faceted concept that may not have a precise definition. It fell among the notions of well-being, satisfaction, and happiness. QOL in the aspect that is of interests of policy makers and urban planners shall be named as quality of urban life (QOUL). Some examples of QOUL indicators were employment rates, housing and neighborhood satisfaction, etc. (Marans 2015).

The six main criteria of the UI GreenMetric Ranking are based on the practices for achieving sustainability, which should result in good QOL of stakeholders in Green Campus universities. For example, as part of the setting and infrastructure criterion, universities should provide sufficient green spaces in their campuses. Based on a study in 2013, students perceived green spaces important for the image of the university and as an essential component of the campus environment (Speake et al. 2013). McFarland et al. (2008) also conducted a study that concluded that undergraduate students deem green space as a positive impact to their QOL.

However, a green university initiative may not be the best desire for everyone if the universities are not well-prepared for it. For example, some universities may try to reduce energy consumption by reducing the use of air conditioning. It should be ideal if green buildings, which are designed for natural ventilation and less dependence on air conditioners, are available. Unfortunately, the practice may not be appropriate for buildings that were not designed for it. Without proper design, people in such buildings would not feel very comfortable and happy when the air conditioners are not on. This situation may not result in good QOL for stakeholders in the university.

Therefore, this study aimed to investigate and compare perceptions of stakeholders in a Green Campus university (already in the UI GreenMetric World University Ranking) and a Non-Green Campus university (not yet entered in the ranking system) regarding their QOL. It intended to investigate whether a Green Campus university, which has performed well according to the six major criteria of the UI GreenMetric World University Ranking, truly posed positive impacts on QOL of stakeholders in the universities as compared to the one not yet ranked. Universities could apply the results to shape their sustainable practices to make them appropriate with their existing conditions. For Non-Green Campus universities, the results would be useful for them to consider entering or prepare themselves for the ranking system.

## Methods

### Study setting

Study populations included institutional units of Mahidol University (MU) and King Mongkut’s Institute of Technology Ladkrabang (KMITL) representing Green Campus and Non-Green Campus universities, respectively. Their international units are named Mahidol University International College (MUIC) and KMITL International College (KMITL-IC), respectively. MUIC and KMITL-IC have similar geographical locations. MUIC is located in Nahhon Pathom just beyond the western border of Bangkok, while KMITL-IC is located in Ladkrabang District, one of the most eastward districts of Bangkok. Both campuses are considered to be located in a suburban area.

In 2013, MU was ranked top among Thai universities (world’s 31st) in the UI GreenMetric World University Ranking. The university was ranked second of Thailand (world’s 71st) in 2014. MUIC was also one of the pilot academic units at MU to implement Green Campus initiatives. Due to its long participation and performance in UI GreenMetric World University Ranking, MUIC was selected to be the representative of Green Campus universities for this comparison.

In order to investigate a comparable institution from a Non-Green Campus, KMITL-IC was chosen to be the representative institution for this study. In 2014, by the time this study was conducted, KMITL had not yet entered the ranking system. It should be noted that, by the end of 2014, KMITL submitted its data to UI for the first time to participate in the ranking. The university was later ranked 12th of Thailand (world’s 258th out of 360 participating universities), which was still quite far below MU. Therefore, compared to MU, KMITL did not perform well according to the ranking criteria and was a good representative for Non-Green Campus universities for this study.

Study populations from these two colleges also reflected a greater variety concerning nationalities of stakeholders (e.g., multi-nationality students) compared to traditional Thai academic units. This diversity would allow the comparison of perspectives between those of Thai and foreign stakeholders. Stakeholders, which include students, lecturers, and staff members at these institutions, were set as the study population.

### Sample size

The necessary sample population from MUIC and KMITL-IC is calculated by using Taro Yamane’s rule. Yamane provided a simplified formula to calculate sample sizes, where *n* is the sample size, *N* is population size, and *e* is level of precision as in (1) (Yamane 1967):

1 $$n = \frac{N}{{1 + Ne^{2} }}$$

Using the Yamane’s rule, the sample size of this study is considered appropriate by 95 % confidence level with a precision rate of ±5 %. The degree of maximum variability is 0.5. The minimum size of sample populations was 524 including 364 from MUIC and 160 from KMITL-IC.

### Questionnarie design

The questionnaire consisted of two parts. Part I was designed to collect demographic information of the respondents. The questions were aimed to ask about the age, gender, status, and study level. The responses were measured using multiple-choice questions. Part II of the questionnaire consisted of 15 questions designed to ask about the aspects related to Green Campus and the perception of the respondents regarding QOL. These questions were mainly derived from the six categories of the UI GreenMetric survey, which consists of setting and infrastructure, energy and climate change, waste management, water usage, transportation, and environmental education. Each category includes questions designed to collect data that reflects the status of ranked universities. To ensure correct understanding and interpretation of the questions, all questions were listed in English with Thai translation provided underneath each question.

For example, the respondents were asked whether their universities provide enough green space to support high QOL. The amount of green space on campus is one of the subcategories under ‘Setting and Infrastructure’ of the UI GreenMetric survey. This is also agreeable with McFarland’s (2014) conclusion that available green space is important for good QOL. The responses were measured using a five-point Likert scale. The alternative items were assigned from 5 (strongly agree) to 1 (strongly disagree). The total mean scores of responses received from Part II were expected to reflect perceived QOL of respondents resulted from sustainability management according to the UI GreenMetric criteria.

Prior to the data collection, the questionnaire was tested for its validity. A pool of 45 questions designed to ask about the aspects related to Green Campus and the perception of the respondents regarding QOL (Part II of the questionnaire) was initially established. The questions were reviewed by a panel of three experts of the Southeast Asian Center for Urban Sustainability (SEACUS), who are expertized in the field of sustainability study to select the best in terms of clarity of the questions, accuracy of the opinion measured, and interpretability. The questions were revised based on comments received. The total number of questions was eventually reduced to 15 questions as stated earlier. These questions were again reviewed and approved by the experts before the questionnaire was used for the study. The Cronbach Alpha Method was applied to ascertain the reliability of the responses for the items based on the five-point Likert scale. The reliability test was conducted with a total of 20 respondents from other universities not included in the sample. The result yielded Cronbach’s alpha of 0.70, which showed adequate degree of reliability (Radhakrishna 2007).

### Data collection

Two channels were used for questionnaire distribution. The first channel was by distributing hard copy questionnaires manually at the two colleges. A web-based survey tool (Survey Monkey^®^) was used as the second channel. A link to the web-based survey was sent through MUIC and KMITL-IC webmail systems with a reminder email sent 1 week after. No incentive was given for the respondents to complete the questionnaires. The study received responses from 530 respondents during 2-week data collection period including 370 responses from MUIC and 160 responses from KMITL-IC. The amount of samples collected complied with the expected sample group size.

### Data analysis

Data collected were entered into an Excel file and analyzed with IBM SPSS Statistics for Microsoft Windows Version 13.0. Percentage and frequency were used to analyzed and present demographic information of the respondents. Arithmetic mean was used to calculate the average level of responses in the five-point Likert scale. The calculated mean scores were subsequently used to conduct T-test and one-way ANOVA (F-test) to find the difference in the attitudes between stakeholders in Green and Non-Green Campuses. Determination of significance level was set at 0.05.

## Results and discussion

### Part I: respondents’ demographic conditions and perceived QOL

Demographic information of the respondents was collected and presented in Table 1. These sets of information are presented in this section because studies in the past regarding relationship between respondents’ demography and level of environmental awareness showed some controversial findings. Abdul-Wahab and Abdo (2010) investigated the effects of demographic factors including gender, age, and education level on the environmental awareness of Omani citizens. The study found that males had a higher level of knowledge about environmental issues than females. Males were also more environmentally concerned and tended to engage in more environmental behaviors than females. Younger and more educated people tended to be more concerned about the environment than older and less educated people. This finding was, however, somewhat contradictory with a study conducted in Malaysia, which concluded that higher age and level of education resulted in better environmental awareness and attitude (Aminrad et al. 2011).

**Table 1:** Demographic information of the respondents

Demographic conditions	MUIC		KMITL-IC	
	Number	Percentage	Number	Percentage
Gender				
Male	144	38.9	88	55
Female	226	61.1	72	45
Age (years)				
≤20	146	39.5	58	36.3
21–29	115	31.1	69	43.1
≥30	109	29.4	33	20.7
Status				
Student	261	71.4	152	95
Staff	106	28.7	8	5
Student study level				
Undergraduate	259	98.1	107	70.4
Graduate student	5	1.9	45	29.6

Omoogun and Odok (2013) also reported a contradictory finding. Their study indicated that there was a significant influence of gender and environmental awareness on attitude of people towards forest conservation. Males were considered as primary destroyers of the forest, while females were deemed as secondary users of the forests. However, Shivakumara et al. (2015) investigated the effect of gender on environmental awareness of past-graduate students in two universities in India. The study concluded that gender has no significant effect on environmental awareness of the post-graduate students.

T-test analyses were conducted to investigate whether opinions about perceived QOL on campus were significantly different by demographic factors of the respondents of this study. As illustrated in Table 2, the combined mean scores of responses received from male and female respondents of MUIC were 4.05 and 4.04, respectively. The results showed that male respondents seemed more satisfied with sustainability management aspects at their campus and had slightly better perceived QOL. The difference between the mean scores was minimal. A T-test analysis indicated that the difference was not statistically significant. Similar results were obtained from KMITL-IC respondents. The combined mean scores received from male and female respondents of KMITL-IC were 3.91 and 3.89, respectively. A T-test analysis also showed that the difference between the mean scores was not statistically significant.

**Table 2:** The comparison of mean scores, T-test values and p-values between male and female respondents regarding their perceived QOL

Item	Questions^b^	MUIC				KMITL-IC			
		Male	Female	T-test value	p-value	Male	Female	T-test value	p-value
Q1	Environmental management is important for a university’s campus	4.52	4.49	0.48	0.63	4.17	4.28	0.53	0.39
Q2	You are satisfied with environmental management of your university	4.03	3.91	1.43	0.15	3.56	3.47	0.45	0.56
Q3	University’s available green space is important for you	4.26	4.24	0.30	0.77	4.18	4.21	0.99	0.82
Q4	Your university provides enough green space to support a high quality of life	3.98	4.00	−0.18	0.86	3.60	3.63	0.88	0.89
Q5	Energy saving is very important practice for your university	4.06	4.13	−0.84	0.40	3.95	4.03	0.09	0.56
Q6	University’s energy saving practices does support high quality of life	3.95	4.15	−2.19	0.03^a^	4.02	4.10	0.69	0.52
Q7	Climate change mitigation programs (greenhouse gas emission reduction) are very important practices for your university	3.92	3.79	1.32	0.19	3.85	3.86	0.41	0.95
Q8	Waste management (example waste separation, waste reduction) is very important practices for your university	4.18	4.18	0.57	0.99	4.06	4.04	0.34	0.91
Q9	University’s waste management (example waste separation, waste reduction) does support a high quality of life	4.12	4.09	0.35	0.73	4.03	3.94	0.28	0.47
Q10	University’s water management (water saving) does support a high quality of life	4.10	4.05	0.59	0.56	4.11	3.96	0.71	0.22
Q11	University’s transportation condition (amount of traffic, availability of public transportation, etc.) does support a high quality of life	3.85	3.84	0.11	0.92	3.92	3.75	0.94	0.25
Q12	University’s environmental education (academic courses and activities related to environmental) does support a high quality of life	4.21	4.14	0.78	0.44	4.09	4.07	0.21	0.87
Q13	You are satisfied with overall quality of your life on campus	4.05	4.05	0.00	1.00	3.64	3.54	0.03	0.45
Q14	If you are a university applicant, Green Campus status would be one of your selection criteria	3.84	3.74	1.17	0.24	3.85	3.74	0.74	0.56
Q15	University’s Green Campus does support a high quality of life on campus	3.73	3.86	−1.41	0.16	3.74	3.76	0.78	0.86
	Combined mean scores	4.05	4.04	0.27	0.80	3.91	3.89	0.26	0.68

^a^The mean scores are significantly different when p-value is at or below 0.05

^b^The responses were measured using a five-point Likert scale. The alternative items were assigned from 5 (strongly agree) to 1 (strongly disagree). Respondents were instructed to choose alternative items depending on how they were agree with the statements. For example, the respondent shall select ‘5’ if they strongly agree with the statements, while selecting ‘1’ if they least agree with the statements

Table 3 illustrates the differences in mean scores and T-test analysis results conducted to compare results received between study levels of the respondents. The combined mean scores received from undergraduate and graduate students of MUIC were 3.96 and 3.93, respectively. The difference between the mean scores was not statistically significant. There was no different between the mean scores received from undergraduate and graduate students of KMITL-IC. The mean scores were 3.90 from both groups of the respondents. Therefore, there was no significant difference between the mean scores of responses of both MUIC and KMITL-IC by gender and study level.

**Table 3:** The comparison of mean scores, T-test values and p-values between undergraduate and post-graduate students regarding their perceived QOL

Item	Questions^b^	MUIC				KMITL-IC			
		Undergraduate	Postgraduate	T-test value	p-value	Undergraduate	Postgraduate	T-test value	p-value
Q1	Environmental management is important for a university’s campus	4.43	4.44	−0.02	0.98	4.24	4.14	0.74	0.46
Q2	You are satisfied with environmental management of your university	3.92	3.89	0.29	0.77	3.49	3.65	−1.08	0.28
Q3	University’s available green space is important for you	4.13	4.17	−0.36	0.72	4.21	4.06	1.19	0.24
Q4	Your university provides enough green space to support a high quality of life	4.00	3.93	0.63	0.53	3.60	3.53	0.41	0.68
Q5	Energy saving is very important practice for your university	4.04	3.93	1.06	0.29	3.94	3.98	−0.28	0.78
Q6	University’s energy saving practices does support high quality of life	3.95	3.89	0.46	0.64	4.05	4.02	0.23	0.82
Q7	Climate change mitigation programs (greenhouse gas emission reduction) are very important practices for your university	3.64	3.77	−0.97	0.33	3.79	4.06	−1.74	0.08
Q8	Waste management (example waste separation, waste reduction) is very important practices for your university	4.09	4.02	0.61	0.54	4.05	4.08	−0.23	0.82
Q9	University’s waste management (example waste separation, waste reduction) does support a high quality of life	3.92	3.96	−0.38	0.70	3.99	3.98	0.08	0.94
Q10	University’s water management (water saving) does support a high quality of life	3.87	3.96	−0.81	0.42	4.09	3.98	0.78	0.44
Q11	University’s transportation condition (amount of traffic, availability of public transportation, etc.) does support a high quality of life	3.89	3.70	1.16	0.25	3.85	3.92	−0.41	0.69
Q12	University’s environmental education (academic courses and activities related to environmental) does support a high quality of life	4.16	3.96	1.64	0.10	4.16	3.94	1.50	0.14
Q13	You are satisfied with overall quality of your life on campus	3.95	3.94	0.11	0.91	3.55	3.67	−0.87	0.38
Q14	If you are a university applicant, Green Campus status would be one of your selection criteria	3.66	3.72	−0.50	0.62	3.78	3.84	−0.39	0.70
Q15	University’s Green Campus does support a high quality of life on campus	3.82	3.70	0.932	0.35	3.75	3.69	0.35	0.73
	Combined mean scores	3.96	3.93	0.63	0.53	3.90	3.90	0.02	1.00

^a^The mean scores are significantly different when p-value is at or below 0.05

^b^The responses were measured using a five-point Likert scale. The alternative items were assigned from 5 (strongly agree) to 1 (strongly disagree). Respondents were instructed to choose alternative items depending on how they were agree with the statements. For example, the respondent shall select ‘5’ if they strongly agree with the statements, while selecting ‘1’ if they least agree with the statements

As presented in Table 4, the mean scores of KMITL-IC respondents in age levels less than 20 years old, 21–29 years old, and more than 30 years old were 3.86, 3.93, and 3.88, respectively. An F-test analysis showed that the difference of mean scores was not statistically significant (F-test value 0.55, p-value 0.58). However, the mean scores of responses received from MUIC showed significant difference of responses by ages of respondents. The mean scores of MUIC respondents in age levels less than 20 years old, 21–29 years old, and more than 30 years old were 3.91, 4.01, and 4.33, respectively. The results implied that older respondents were more satisfied with sustainability management aspects at their campus and had higher perceived QOL.

**Table 4:** The comparison of mean scores, F-test values and p-values among different age levels of respondents regarding their perceived QOL

Item	Questions^b^	MUIC					KMITL-IC				
		<20 years	21–29 years	>30 years	F-test value	p-value	<20 years	21–29 years	>30 years	F-test value	p-value
Q1	Environmental management is important for a university’s campus	4.44	4.44	4.71	5.46	0.01^a^	4.10	4.32	4.07	1.60	0.21
Q2	You are satisfied with environmental management of your university	3.92	3.87	4.16	3.28	0.04^a^	3.45	3.58	3.43	0.44	0.64
Q3	University’s available green space is important for you	4.14	4.22	4.49	6.11	0.00^a^	4.09	4.24	4.36	1.12	0.33
Q4	Your university provides enough green space to support a high quality of life	4.01	3.90	4.11	1.60	0.20	3.64	3.57	3.79	0.30	0.74
Q5	Energy saving is very important practice for your university	3.92	4.08	4.47	13.70	0.00^a^	3.84	4.05	4.21	1.76	0.18
Q6	University’s energy saving practices does support high quality of life	3.90	3.97	4.53	17.76	0.00^a^	3.97	4.11	4.07	0.74	0.48
Q7	Climate change mitigation programs (greenhouse gas emission reduction) are very important practices for your university	3.65	3.84	4.18	8.30	0.00^a^	3.71	4.00	3.57	2.39	0.10
Q8	Waste management (example waste separation, waste reduction) is very important practices for your university	4.03	4.13	4.54	10.19	0.00^a^	3.90	4.17	3.93	2.14	0.12
Q9	University’s waste management (example waste separation, waste reduction) does support a high quality of life	3.87	4.10	4.51	18.55	0.00^a^	4.09	3.91	4.14	1.21	0.30
Q10	University’s water management (water saving) does support a high quality of life	3.86	4.09	4.39	11.81	0.00^a^	4.12	3.99	4.07	0.50	0.61
Q11	University’s transportation condition (amount of traffic, availability of public transportation, etc.) does support a high quality of life	3.68	3.89	4.02	3.60	0.03^a^	4.12	4.03	4.21	0.37	0.69
Q12	University’s environmental education (academic courses and activities related to environmental) does support a high quality of life	3.93	4.16	4.58	16.20	0.00^a^	1.62	1.48	1.57	0.47	0.63
Q13	You are satisfied with overall quality of your life on campus	3.91	4.03	4.33	9.57	0.00^a^	3.55	3.63	3.57	0.16	0.86
Q14	If you are a university applicant, Green Campus status would be one of your selection criteria	3.72	3.72	4.02	3.77	0.02^a^	3.93	3.75	3.36	2.61	0.08
Q15	University’s Green Campus does support a high quality of life on campus.	3.75	3.80	3.94	1.26	0.29	3.72	3.76	3.79	0.04	0.96
	Combined mean scores	3.91	4.01	4.33	40.03	0.00^a^	3.86	3.93	3.88	0.55	0.58

^a^The mean scores are significantly different when p-value is at or below 0.05

^b^The responses were measured using a five-point Likert scale. The alternative items were assigned from 5 (strongly agree) to 1 (strongly disagree). Respondents were instructed to choose alternative items depending on how they were agree with the statements. For example, the respondent shall select ‘5’ if they strongly agree with the statements, while selecting ‘1’ if they least agree with the statements

The results showed that the respondents showed no significant differences in perceived QOL by most demographic factors. This is an exception of MUIC respondents, which showed significant different responses varied by age. Based on these results, it was expected that comparison of perceived QOL by Green and Non-Green Campus respondents presented in Part II of the study was not altered by demographic factors.

### Part II: Green Campus and perceived QOL

Table 5 presents the mean scores of responses of MUIC and KMITL-IC respondents regarding sustainability aspects covered in the UI GreenMetric World University Ranking and their perceived QOL. The overall mean scores of MUIC and KMITL-IC respondents were 4.04 and 3.90, respectively. The mean scores were significantly different per the result of a T-test analysis. The result implied that MUIC respondents have higher perceived QOL compared to KMITL-IC respondents. The question asking about the importance of green space for high QOL (Question 4) also confirmed a finding presented by McFarland et al. (2008) that available green space is important for good QOL.

**Table 5:** The comparison of mean scores, T-test values and p-values between MUIC and KMITL-IC responses regarding their perceived QOL

Item	Questions^b^	Mean		T-test value	p-value
		MUIC	KMITL-IC		
Q1	Environmental management is important for a university’s campus	4.50	4.22	3.97	0.00^a^
Q2	You are satisfied with environmental management of your university	3.95	3.52	5.20	0.00^a^
Q3	University’s available green space is important for you	4.25	4.19	0.77	0.44
Q4	Your university provides enough green space to support a high quality of life	3.99	3.61	4.10	0.00^a^
Q5	Energy saving is very important practice for your university	4.11	3.99	1.58	0.12
Q6	University’s energy saving practices does support high quality of life	4.07	4.06	0.20	0.85
Q7	Climate change mitigation programs (greenhouse gas emission reduction) are very important practices for your university	3.84	3.86	−0.17	0.86
Q8	Waste management (example waste separation, waste reduction) is very important practices for your university	4.18	4.05	1.66	0.10
Q9	University’s waste management (example waste separation, waste reduction) does support a high quality of life	4.10	3.99	1.42	0.16
Q10	University’s water management (water saving) does support a high quality of life	4.07	4.04	0.31	0.75
Q11	University’s transportation condition (amount of traffic, availability of public transportation, etc.) does support a high quality of life	3.84	3.84	−0.04	0.97
Q12	University’s environmental education (academic courses and activities related to environmental) does support a high quality of life	4.16	4.08	1.03	0.30
Q13	You are satisfied with overall quality of your life on campus	4.05	3.59	6.30	0.00^a^
Q14	If you are a university applicant, Green Campus status would be one of your selection criteria	3.79	3.78	0.06	0.95
Q15	University’s Green Campus does support a high quality of life on campus	3.81	3.75	0.72	0.47
	Combined mean scores	4.04	3.90	3.96	0.00^a^

^a^The mean scores are significantly different when p-value is at or below 0.05

^b^The responses were measured using a five-point Likert scale. The alternative items were assigned from 5 (strongly agree) to 1 (strongly disagree). Respondents were instructed to choose alternative items depending on how they were agree with the statements. For example, the respondent shall select ‘5’ if they strongly agree with the statements, while selecting ‘1’ if they least agree with the statements

The results emphasized that being a Green Campus University by applying criteria set in the UI GreenMetric World University Ranking does pose a positive impact on perceived QOL. Respondents in a Green Campus university provided statistically significant responses indicating that they have better perceived QOL than respondents in a Non-Green Campus university.

## Conclusions

The study was conducted to compare stakeholders’ perception about perceived QOL between those in Green and Non-Green Campus universities. Study results clearly showed that MUIC respondents, whose campus better complied with sustainability practices listed in the UI GreenMetric World University Ranking, were more satisfied with sustainability aspects at their campus and have better perceived QOL. This was demonstrated through the mean scores of a set of questions derived from the six major criteria of the UI GreenMetric World University Ranking designed to measure perceived QOL on campus. The results were statistically significant by a T-test analysis. The instrument used in the study had adequate reliability with Cronbach’s alpha of 0.70.

Responses received from the study showed no significant difference by demographic conditions (i.e., gender, age, and study level) of the respondents. This result strengthened the conclusion that respondents of the Green Campus university were more satisfied with sustainability management and QOL at their campus regardless of demographic factors. It should be noted that this finding was in line with a study of Shivakumara et al. (2015), which concluded that gender has no significant effect on environmental awareness. However, the finding was also contradictory with the results of some past studies, which concluded that some demographic factors such as gender, age, and level of education influenced the level of environmental awareness and attitude (Abdul-Wahab and Abdo 2010; Aminrad et al. 2011; Omoogun and Odok 2013).

The results obtained seemed not surprising especially for the general public. A university that performed better in the UI GreenMetric World University Ranking was expected to offer better sustainability management and QOL. However, any measures to be implemented according to the UI GreenMetric World University Ranking criteria such as energy saving and reduction in the use of private vehicles should be done with careful considerations to avoid unnecessary compromising of good QOL of stakeholders in the universities. For example, limiting the use of air conditioning system may be implemented only if the building can facilitate natural ventilation. Reduction in the use of private vehicles should be done along with a sufficient on-campus public transportation system.

It can be concluded that universities should promote and try to adopt the criteria set in the UI GreenMetric World University Ranking for their campuses. Being a green university would increase more positive perception of stakeholders about campus QOL. The initiative would also help raise better awareness about sustainability for universities’ stakeholders. The universities could also use the Green Campus initiative for marketing purposes for student recruitment. Green Campus initiatives seem to be ones of the prominent channels to promote and support world sustainability.
